# A Machine Learning Approach for Predicting Synergism of Mixtures of Compounds in Treating Cancers

**DOI:** 10.3390/ijms27188345

**Published:** 2026-09-19

**Authors:** Azar Shamloo, Jack Tuszynski, Yun Tam, Chih-Yuan Tseng

**Affiliations:** 1 Department of Physics, University of Alberta, 11335 Saskatchewan Dr NW, Edmonton, AB T6G 2M9, Canada; jackt@ualberta.ca; 2 Department of Mechanical and Aerospace Engineering, Politecnico di Torino, Corso Duca degli Abruzzi, 24, 10129 Turin, Italy; 3 SinoVeda Canada Inc., 1059 Tory Road NW, Edmonton, AB T6R 3A5, Canada; ytam@sinoveda.com

**Keywords:** ensemble stacking machine learning method, pairwise-compound quantitative structure–activity relationship (pQSAR), combination therapy, cancer treatment

## Abstract

Combination therapy is a common strategy in cancer treatment to improve efficacy, minimize side effects, and overcome drug resistance. However, the vast number of possible drug pairings makes experimental screening both time-consuming and costly. Drug interactions can result in synergy and antagonism, emphasizing the need for efficient predictive models. In this study, we develop a pairwise-compound quantitative structure–activity relationship (pQSAR) framework based on an ensemble stacking machine learning strategy to enhance prediction of empirically defined combination-effect classes using molecular and PK-related descriptors. Our approach leverages molecular descriptors along with key pharmacokinetic properties, the estimated accumulated drug concentration over 24 h (AUC), bioavailability (Fb) and volume of distribution at steady state (Vdss) to train the model on pairwise drug combinations. Building upon this foundation, we develop an ensemble stacking classification model that integrates multiple ML algorithms, providing a more accurate and robust prediction of drug synergy. Using the ALMANAC drug dataset across 8 cell lines from 4 different cancer types, our model achieves an accuracy exceeding 0.88 in predicting the effects of drug pairs. These results indicate that the proposed framework can capture predictive patterns associated with experimentally defined drug-combination effect classes in the evaluated dataset.

## 1. Introduction

Drug combinations, also referred to as combinatorial therapies, are commonly used to treat complex diseases, particularly cancers [1,2], which often involve multiple underlying mechanisms [3]. The main advantage of combination therapy is that simultaneously targeting multiple molecular pathways in cancer cells can enhance treatment effectiveness [1,2]. As a result, compared to monotherapies (single-drug treatments), which may have limited efficacy, drug combinations have been shown to improve therapeutic outcomes [1,2], reduce side effects [4], and help overcome drug resistance in cancer treatment [5]. However, the simultaneous administration of multiple drugs can sometimes lead to adverse effects [6]. For example, while Hodgkin lymphoma treatments have achieved an impressive average 10-year relative survival rate of 85% for patients aged 15 to 54 years [7], older patients are at an increased risk of developing acute myelogenous leukemia [8]. Additionally, survival rates for most metastatic cancers remain disappointingly low. Beyond issues of efficacy and side effects in both monotherapies and combination therapies, the development of anti-cancer drugs remains prohibitively expensive and time-consuming. Repurposing existing therapeutic agents for use in combination therapies presents a potential solution to these challenges [9]. The ultimate goal of precision medicine is to tailor therapies that enhance patient survival while minimizing adverse side effects. This can be achieved by matching patients with the most suitable drug combinations based on their molecular and clinical profiles [10]. Identifying optimal drug combinations solely through experimental screening remains a challenging and resource-intensive task; the methods used to address this challenge are discussed in the following Section 1.2.

### 1.1. Computational Approaches for the Prediction of Drug Combination Synergy

As discussed above, identifying optimal drug combinations through experimental screening is a complex and resource-intensive process due to the vast number of possible permutations. Therefore, there is a critical need for advanced computational tools that can efficiently predict effective and synergistic drug combinations for cancer treatment [11]. Previous studies have explored a wide range of computational approaches to predict anticancer drug synergy. Early methods were largely hypothesis-driven, relying on systems biology frameworks, signaling pathways, and biomolecular or protein–protein interaction networks to infer potential synergistic effects. While these approaches offered biological interpretability, they were often limited by small, incomplete, and noisy datasets, as well as strong assumptions that restricted their generalizability across drugs, cancer types, and cell lines [12,13,14].

With the increasing availability of large-scale drug screening data, data-driven methods became more prominent. Recent advancements in precision medicine have increasingly integrated machine learning (ML) with biomedical research, enabling data-driven approaches to disease assessment and treatment. As a subset of artificial intelligence, ML excels at extracting meaningful patterns from large-scale datasets, making it a valuable tool for drug discovery and oncology research [15]. Traditional machine learning models such as Random Forests [16], gradient boosting [17], extremely randomized trees [18], and tensor factorization [19] leveraged engineered drug and cell line features to learn synergy patterns from experimental data. Several studies have demonstrated the potential of ML in predicting synergistic drug interactions. Celebi et al. developed an in-silico framework that integrates multi-omics and drug data to predict synergistic anticancer drug combinations, demonstrating that XGBoost models leveraging biologically relevant features can efficiently prioritize promising dual therapies across diverse cell lines [20]. El-Hafeez et al. have introduced machine learning frameworks that classify and predict effective cancer drug combinations, achieving improved accuracy in synergy predictions [21]. Zhou et al. developed a suite of ML models that significantly enhance the predictive performance of drug synergy assessments [22]. It was demonstrated that pharmacological synergy or antagonism is influenced by target-related network topology, suggesting that designing synergistic drug combinations based on network properties could be advantageous [23].

More recently, deep learning models have been introduced to automatically capture complex, high-dimensional relationships among molecular structures, gene expression profiles, and drug responses. Models such as DeepSynergy [14], AuDNNsynergy [24], PRODeepSyn [25], and TranSynergy [26] demonstrated improved predictive performance, with some incorporating graph neural networks or transformer architectures to exploit biological network information. In a large-scale community challenge, multiple computational methods, primarily based on machine learning models integrating drug features and cell line molecular profiles, were benchmarked for anticancer drug synergy prediction, demonstrating that data-driven approaches can achieve performance comparable to experimental reproducibility [27]. Furthermore, a comprehensive review categorized state-of-the-art ML approaches for analyzing personalized drug combination therapies, highlighting their advantages in precision medicine [28]. These advancements underscore the transformative potential of machine learning in drug discovery, paving the way for more effective and personalized cancer treatment strategies.

### 1.2. Current Approaches for the Prediction of Drug Combination Synergy

In drug–drug combinations, each drug may act on its own receptor or target a shared receptor. Research in this field has predominantly aimed at characterizing synergy, quantifying dose–response relationships and determining whether specific drug pairs exhibit synergistic effects based on established synergy models and experimental findings [29]. Since Loewe introduced the Loewe additive model in 1926 to describe synergistic drug interactions, numerous researchers have contributed to the study of drug combinations [30]. Loewe later developed the Loewe additive equation to assess whether a specific drug combination would produce a synergistic effect [31]. The Bliss Independence model was first introduced by C.I. Bliss in 1939 and is based on probabilistic theory. This model assumes that if two drugs act independently, their combined effect can be predicted by the multiplication of their individual probabilities of action [32].

Chou and Talalay [33,34,35,36] proposed the median-effect combination index (CI-Isobologram) and the dose-reduction index formula [33] to quantitatively assess drug interactions. According to this model, a CI value of less than 1 indicates synergy, CI = 1 represents an additive effect, and CI greater than 1 suggests antagonism. Moreover, the Highest Single Agent (HSA) model assumes that the expected effect of a drug combination is equivalent to the effect of the most potent individual drug at the given dose within the combination [37]. This concept implies that a synergistic interaction occurs only if the combination achieves an effect greater than what either drug can accomplish alone. However, in many preclinical drug combination studies, even a drug combined with itself can surpass the HSA threshold, highlighting the limitations of this model in accurately distinguishing true synergy from additive effects. Furthermore, Greco introduced the universal response surface approach (URSA), an innovative framework for analyzing drug interactions [38]. URSA models the combined effects of drugs across varying concentrations, allowing for a more precise assessment of synergy, additivity, or antagonism. This method provides a flexible and quantitative way to analyze drug interactions beyond traditional models.

Consequently, Yadav et al. introduced the Zero Interaction Potency (ZIP) model to quantify drug interactions by comparing the observed potency shift in drug combinations relative to a non-interacting reference model [39]. ZIP assesses whether two drugs interact synergistically, antagonistically, or additively based on their expected independent effects. This model overcomes some of the limitations of the previous ones, particularly the incapability to tackle high-throughput results, by combining the advantages of Loewe and Bliss [39].

Although interest in this approach continues to grow, an accepted standardized model has not yet been developed. Several software tools have been developed to integrate and standardize the results of drug combination screening studies, such as DrugComb [40] and SYNER-GxDB v2.2.4 [41]. These platforms utilize the SynergyFinder R package 2.0.12 [42] to calculate and compare synergy estimations across various reference models, helping to predict drug combination effects. Additionally, SynPred [43] applies machine learning techniques to forecast drug interactions.

### 1.3. Integrating Conventional Approaches and Machine Learning Methods for Predicting Drug Combination Synergy

As discussed in Paola et al. [44], there are four issues that limit the applications of the above-mentioned methods for predicting drug combination effects. First, there is no consensus on the most appropriate reference model, and second, isobole-based methods such as Loewe, a-score, and CI are limited because they assess synergy only at fixed dose ratios, requiring extensive experiments. Third, Bliss-based metrics can be misleading due to their assumptions about independent action and interpretation of synergy, and fourth, the ZIP score shows poor correlation with other methods, leading to inconsistency in results.

Instead, we proposed and demonstrated a straightforward solution based on empirical rules to identify the degree of synergism using pairwise dose–response heatmap data [44]. Building on this foundation, in this study, our primary goal is to extend our previous ensemble machine learning methodology for predicting Quantitative Structure–Activity Relationship (QSAR) and propose a pairwise-compound QSAR (pQSAR) to predict the effects of drug pair combinations, either synergism or antagonism [45].

Basically, pQSAR is rooted in our previously developed classification ensemble stacking-based ML framework [45]. Following similar arguments in our previous works, potential sources of noise that may influence the results of in vitro activity assays should be taken into consideration. Therefore, these noises may undermine the identification of structure and activity relations. We found out that incorporating a concentration-related pharmacokinetic (PK) property of a compound, the estimated accumulated drug concentration over 24 h (AUC), in the ensemble-based ML models dramatically improves model performance in predicting biological effects of a single compound [45]. However, to predict effects of drug pairs, we need to address additional questions including “What about other PK properties?” and “What types of concentration-related PK properties shall we consider?” Therefore, we conducted a thorough investigation to explore the impacts of different PK properties on model performance. Note that the PK properties of compounds typically refer to 4 types of processes happened to the compounds in the body: absorption, distribution, metabolism, and excretion. Among these four processes, we can roughly group metabolism and excretion into “elimination” phases, which “remove” compounds from the body. Namely, one may simply consider drugs to undergo two processes, absorption and elimination, when drugs enter the body. Therefore, our investigation focuses on factors associated with absorption and elimination phases in addition to overall concentration-related properties. First, for the overall concentration-related properties, there are three quantities that indicate overall drug concentration changes in the body. They are accumulated drug concentration over 24 h (AUC); bioavailability (Fb), reflecting the extent to which a compound reaches systemic circulation; and volume of distribution at steady state (Vdss), representing the extent of drug distribution between plasma and tissues. Second, factors related to the absorption phase include apparent permeability (Papp), which estimates intestinal absorption based on Caco-2 cell membrane permeability and time to maximum plasma concentration (Tmax). It should be noted that Tmax is influenced by both absorption and elimination processes and is therefore considered a hybrid parameter rather than a purely absorption-related metric. Third, factors related to elimination, they include hepatic intrinsic clearance (CLint), which approximates the hepatic metabolic rate; half-life (t_1/2_), describing the rate at which compounds are eliminated from the body.

In this study, we employed a predictive pQSAR framework to model empirically observed drug combination effects rather than to mechanistically characterize the underlying pharmacodynamic basis of drug synergy.

As we will discuss later, we observed improved predictive accuracy when incorporating concentration-related PK properties including AUC, Vdss, and bioavailability(Fb). Finally, we conducted a comprehensive comparative analysis to evaluate the proposed pQSAR method using the ALMANAC drug dataset for 4 distinct cancer types across eight cancer cell lines, including colorectal cancer (HCT15, HCT116), liver cancer (Huh7), breast cancer (BT-549, HS 578T), and lung cancer (NCI-H460, NCI-H522, A549). The proposed ensemble stacking model integrating RF, GBM, and LR, with LR as the meta-learner, consistently outperformed individual algorithms and the ensemble stacking MLP, achieving accuracy values above 0.88, particularly when concentration-related PK ratio features including AUC, Vdss, and bioavailability (Fb) alongside MACCS fingerprints were included as independent variables. In contrast, absorption- and elimination-related parameters (Papp, Tmax, CLint, and t_1_/_2_) contributed less to model performance.

## 2. Results

### 2.1. Preliminary Evaluation of the Proposed Ensemble Stacking Classification Method

As demonstrated in our previous study [45], ML models trained on datasets encoded with MACCS fingerprints outperformed those using other fingerprinting techniques. We also showed that the effectiveness of ensemble learning strongly depends on the appropriate selection of base learners, as their complementary strengths enhance overall predictive performance while mitigating individual weaknesses. Recent findings further confirm the superiority of ensemble approaches in QSAR classification tasks, where they consistently outperform single models [46].

As will be discussed in Section 4.3, the ensemble stacking developed in this study integrates RF, GBM, and LR as base learners, with LR employed as the meta-learner. Our previous work demonstrated that these algorithms collectively improve predictive accuracy [45]. The ensemble was designed to exploit the strong predictive capabilities of RF and GBM while leveraging LR for optimized decision boundary integration. The selection of base learner algorithms for classification was based on their complementary strengths in handling non-linearity, feature importance, and decision boundary optimization. Before we comprehensively investigated the performance and applicability of the proposed ensemble stacking method, we conducted a preliminary study using data from a common cancer cell line, HCT116.

The proposed ensemble stacking outperforms the individual ML algorithms. A confusion matrix provides a detailed evaluation of classification performance by comparing predicted and actual labels and enabling the calculation of metrics such as precision, recall, and F-score [47].

**Figure 1:**
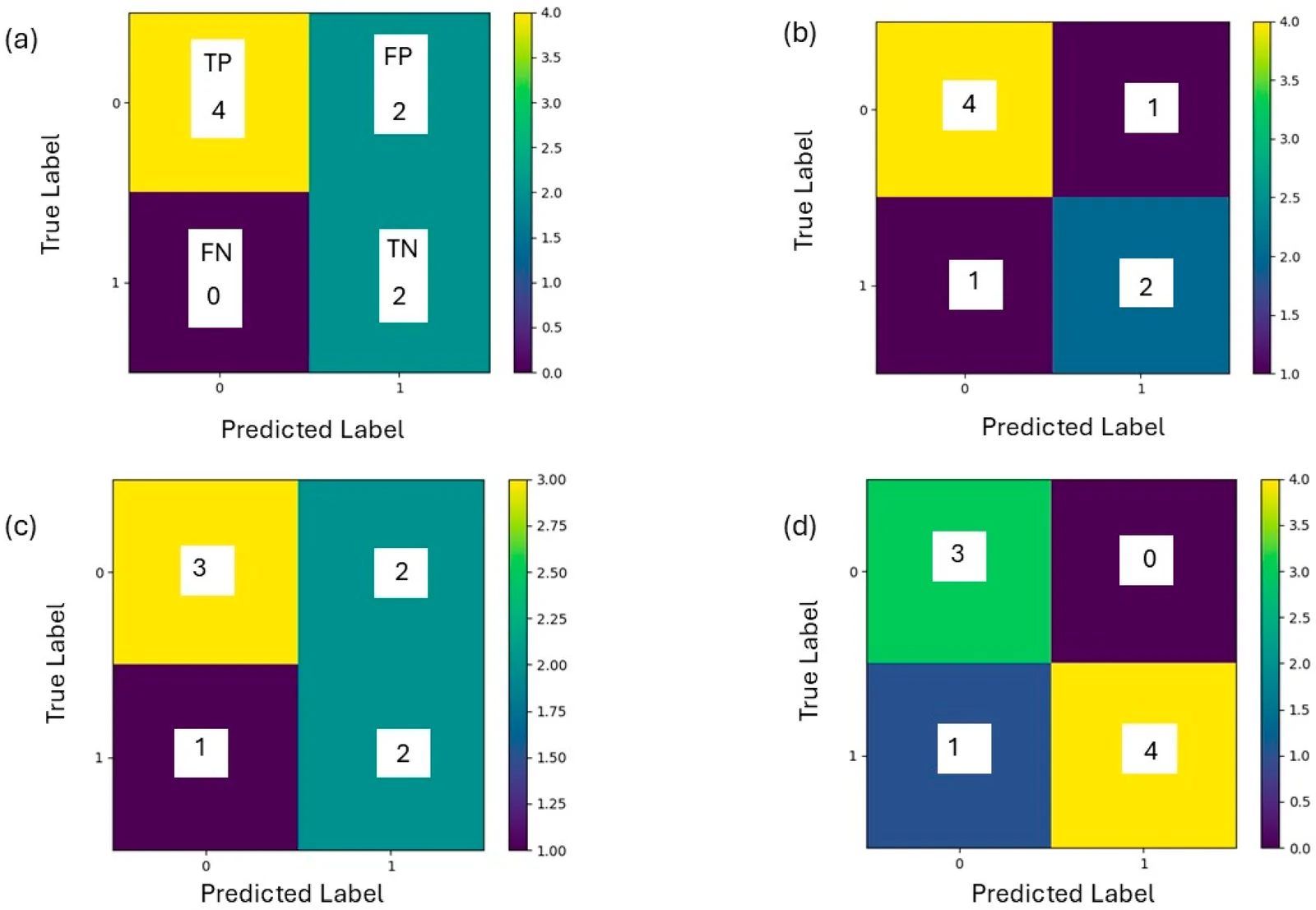
Confusion matrix plots of (**a**) RF, (**b**) GBM, (**c**) LR and (**d**) the proposed ensemble stacking based on the MACCS fingerprint in the validation set of the HCT116 cell line dataset, considering Vdss_1_/Vdss_2_ as independent variables.

Figure 1 presents the confusion matrices for the validation set of the HCT116 cell line dataset considering Vdss_1_/Vdss_2_ as independent variables: RF (a), GBM (b), LR (c), and the proposed ensemble stacking (d). Prior to training, feature standardization was applied to ensure variable comparability and facilitate model convergence. The two activity classes, “synergistic” (class 0) and “antagonistic” (class 1), are shown on the true (y-axis) and predicted (x-axis) labels. TP, FP, FN, and TN (will be defined in Section 4.2.1) are indicated in panel (a), and the color scale reflects the number of samples in each category.

The ensemble stacking achieved the highest combined number of correct predictions (TP + TN = 7), followed by GBM and RF (6 each) and LR (5). Similar trends were consistently observed across the other cell lines.

**Figure 2:**
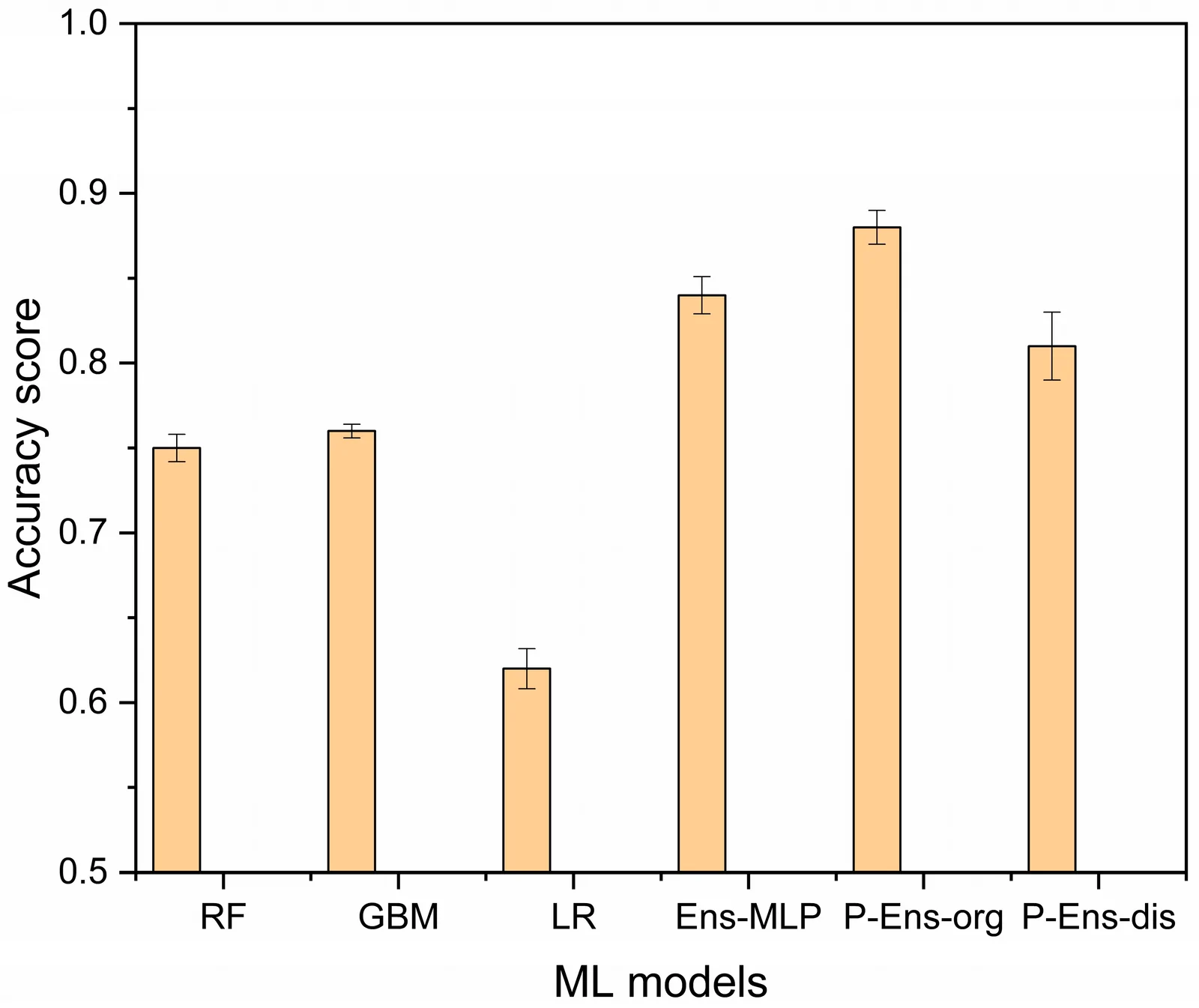
An accuracy score plot for individual and ensemble ML models based on MACCS fingerprint on the testing set of the HCT116 cell line dataset considering AUC_1_/AUC_2_ as independent variables.

**The proposed ensemble stacking method also outperforms other ensemble methods.** Table 1 presents the performance metrics of the individual and ensemble methods on the HCT116 testing set, including RF, GBM, LR, Ensemble stacking with MLP (Ens-MLP), the proposed Ensemble stacking model using the original pair-random split (P-Ens-org), and the proposed Ensemble stacking model using the drug-disjoint train–test split (P-Ens-dis) (will be defined in Section 4.3), with AUC_1_/AUC_2_ used as the independent variable. Among the individual models, GBM achieved the highest performance, followed by RF and LR. The proposed P-Ens-org achieved the highest accuracy (0.88), followed by Ens-MLP (0.84), P-Ens-dis (0.80), GBM (0.76), RF (0.75), and LR (0.62). The proposed P-Ens-org also showed the highest precision, recall, and F1-score among the evaluated models. The lower accuracy of P-Ens-dis reflects the greater difficulty of predicting combinations involving previously unseen drugs; nevertheless, the 0.80 accuracy demonstrates that the proposed Ensemble stacking model maintains good predictive performance under this more stringent drug-disjoint validation, supporting its ability to generalize beyond compounds encountered during training. Figure 2 presents the accuracy scores of the individual and ensemble ML models based on the MACCS fingerprints. In this figure, the proposed Ensemble stacking models using the original pair-random and drug-disjoint splits are denoted as P-Ens-org and P-Ens-dis, respectively, while the Ensemble stacking model with MLP is denoted as Ens-MLP.

### 2.2. Determination of the Optimal Ensemble Stacking Method for Predicting the Effects of Pairwise Drug Combinations

As will be described in Section 4.1.1, we aimed to enhance model performance by incorporating physicochemical and pharmacokinetic (PK) properties of compounds during dataset construction. Specifically, three PK-related categories were considered and systematically evaluated: concentration, absorption, and elimination.

**Figure 3:**
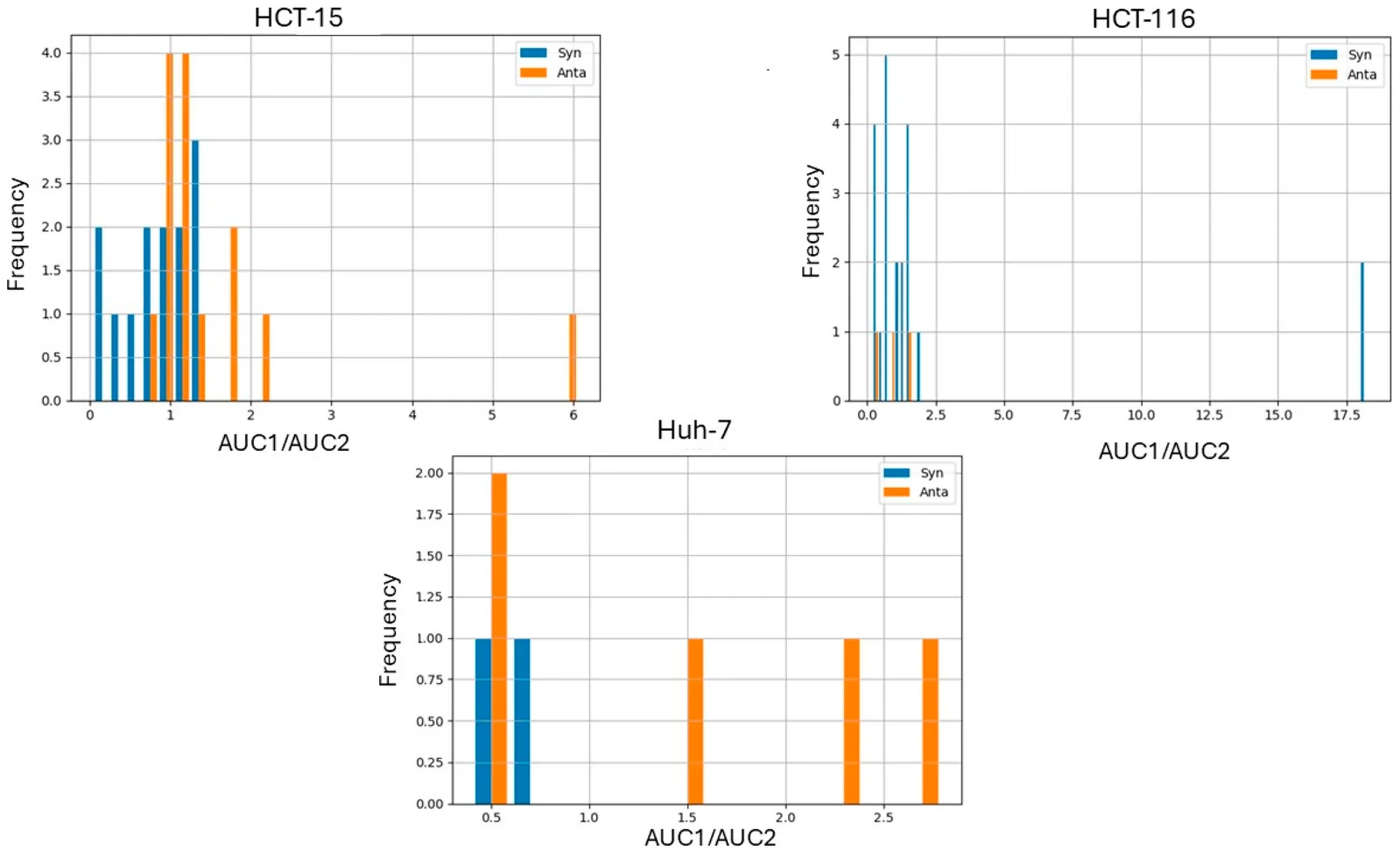
Frequency plots of synergistic and antagonistic pairwise drug combinations across varying AUC1/AUC2 ratios for HCT15, HCT116, and Huh7 cell lines.

**There are no obvious patterns in the PK ratio dataset.** Within the concentration-related category, we included AUC, Fb, and Vdss. For absorption-related properties, we considered Papp and Tmax. For elimination-related properties, CLint and t_1_/_2_ were included. To investigate whether intrinsic patterns in the dataset might account for the model’s predictive performance, we generated frequency plots of synergistic and antagonistic pairwise drug combinations across varying AUC_1_/AUC_2_ ratios. Figure 3 illustrates these distributions for three cell lines: HCT15, HCT116, and Huh7. The plots do not reveal any discernible patterns or separable trends between synergistic and antagonistic combinations. The relatively high predictive accuracy observed in the multivariable model therefore should not be interpreted as evidence that AUC alone is sufficient to predict synergy.

**The model trained using the dataset with concentration-related PK ratios outperforms others.** To further substantiate this hypothesis, we evaluated model accuracy after incorporating the selected pharmacokinetic (PK) properties, including concentration-related parameters (AUC, Fb, and Vdss), absorption-related parameters (Papp and Tmax), and elimination-related parameters (CLint and t_1_/_2_). The analysis was conducted across four cancer types using eight representative cancer cell lines: colorectal cancer (HCT15, HCT116), liver cancer (Huh7), breast cancer (BT-549, HS 578T), and lung cancer (NCI-H460, NCI-H522, A549).

We observed that incorporating PK_1_/PK_2_ or PK_2_/PK_1_ ratios alongside molecular fingerprints as independent variables improves the accuracy of predicting synergistic and antagonistic effects of pairwise drug combinations across eight cancer cell lines. Table 2 summarizes the accuracy scores obtained under three conditions: (i) using PK properties of individual drugs only, (ii) using no PK properties, and (iii) using PK ratio features (PK_1_/PK_2_ or PK_2_/PK_1_). We performed repeated nested cross-validation and estimated confidence intervals for the model performance metrics to further assess the robustness of the observed improvement in predictive performance associated with the PK-derived ratio (will be explained in Section 4.3). The nested cross-validation framework minimizes the potential for optimistic performance estimates arising from model selection and hyperparameter tuning within the same data used for evaluation. The inclusion of confidence intervals and standard deviations for AUC, Vdss and Fb in Table 2 additionally provides an assessment of the variability and uncertainty of the performance estimates across validation folds (will be discussed in Section 3.2). Overall, the results demonstrate consistently higher accuracy when PK ratio features are included, with the observed differences remaining robust across the repeated validation procedure. These findings support the interpretation that the improvement associated with the PK ratios is not solely attributable to a particular train–test split.

**Figure 4:**
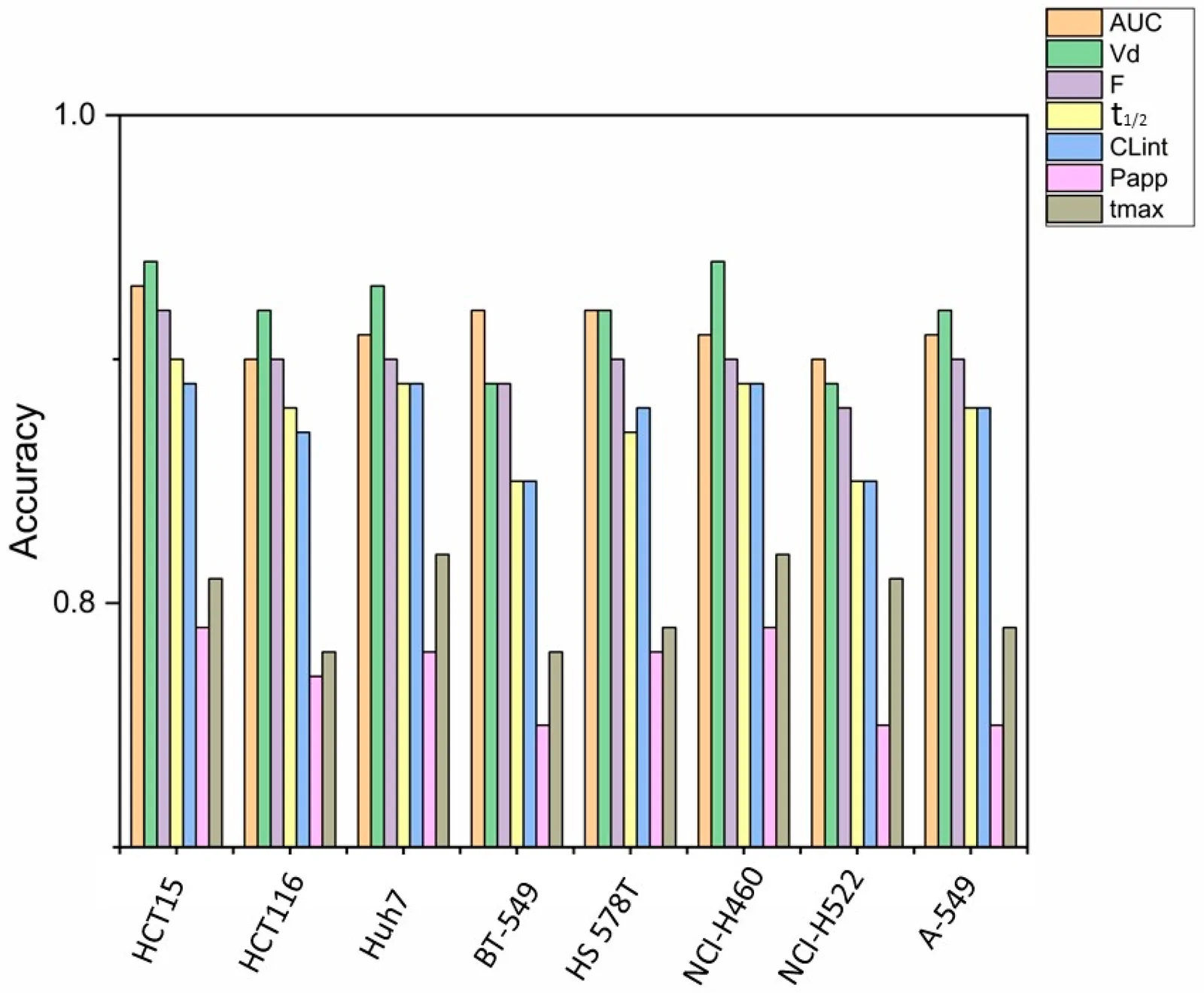
An accuracy score plot incorporating various pharmacokinetic (PK) properties, including AUC, Vdss, Fb, t_1/2_, CLint, Papp, and tmax, alongside molecular fingerprints.

Figure 4 presents the accuracy scores obtained when various pharmacokinetic (PK) properties, including AUC, Vdss, Fb, t_1_/_2_, CLint, Papp, and tmax, are incorporated alongside molecular fingerprints. This Figure presents the predictive accuracy of the complete machine-learning model incorporating the corresponding PK features and MACCS molecular fingerprints, whereas Figure 3 illustrates the distribution of synergistic and antagonistic combinations as a function of the AUC_1_/AUC_2_ ratio alone.

## 3. Discussion

### 3.1. Performance of the Proposed Ensemble Stacking Model

The results in Table 1 indicate that the MACCS-based ensemble stacking model provides the best performance for predicting the effects of pairwise drug combinations in the HCT116 validation set. According to these results, one can conclude that the proposed Ensemble stacking has the best performance among all the other individual methods and Ensemble stacking with MLP.

The improved performance is consistent with the complementary strengths of the selected base learners. RF and GBM effectively capture nonlinear relationships and complex feature interactions, while LR serves as an efficient meta-learner for integrating their predictions into an optimized decision boundary.

### 3.2. Effect of Pharmacokinetic Ratio Features on Model Performance

The absence of obvious patterns in the frequency plots of Figure 3 indicates that the strong predictive performance of the model is not attributable to an inherent bias or obvious structure in the dataset based solely on AUC_1_/AUC_2_ ratios.

A plausible explanation for the improved accuracy observed in Table 2 is that ratio-based features reduce scale heterogeneity among compounds and normalize variability across drugs with widely differing pharmacokinetic (PK) magnitudes, thereby improving the signal-to-noise ratio. As a result, models trained on PK ratio features more effectively capture patterns associated with synergistic and antagonistic interactions.

As shown in Table 2, higher predictive accuracy for the effects of pairwise drug combinations is achieved when concentration-related parameters (AUC, Vdss, and Fb) are included, with the best performance observed for models incorporating AUC and Vdss.

This improvement likely arises because these concentration-related parameters quantitatively capture drug exposure and systemic availability, key determinants of drug action and interaction. This is consistent with prior pharmacokinetic research showing that concentration metrics such as AUC and bioavailability are key determinants of drug exposure and interaction potential in combined therapies [48]. In particular, AUC reflects the extent of drug exposure at the site of action and the overall cellular response to treatment. As an emergent in vivo property, AUC implicitly incorporates factors such as absorption (Fb), elimination (CLint), and distribution (Vdss). Consequently, including AUC enables the model to reduce noise and better identify relationships between compounds and their biological activities.

Moreover, drug synergy and antagonism are inherently interaction-driven phenomena, which depend on the relative pharmacokinetic behavior of two drugs rather than their absolute properties. This relative representation better reflects differences in effective intracellular concentrations and temporal exposure profiles, which are critical determinants of combination effects. Peng et al. reviewed pharmacokinetic drug–drug interactions (DDIs), noting that changes in PK parameters such as AUC, Cmax, and half-life due to co-administered drugs are central to understanding how one drug affects the exposure of another, which in turn influences interaction outcomes [49].

Figure 4 further demonstrates that incorporating concentration-related parameters (AUC, Vdss, and Fb) enhances predictive performance for identifying the effects of pairwise drug combinations, whereas Papp and Tmax exhibit comparatively low predictive power. It is not surprising that Papp exhibits the lowest predictive power, as it is derived from an in vitro permeability assay (e.g., Caco-2 cell models) and therefore does not fully capture the complexity of in vivo physiological processes such as intestinal metabolism, transporter activity, and regional variability in absorption. Consequently, its ability to reflect systemic drug behavior is limited [50].

Similarly, Tmax is not a purely absorption-related parameter. It should be noted that Tmax is influenced by both the rate of absorption and the rate of elimination; specifically, it corresponds to the time point at which the rates of these two processes are equal. As such, Tmax represents a hybrid parameter rather than a direct measure of absorption kinetics alone.

## 4. Materials and Methods

### 4.1. Data Preparation

#### 4.1.1. Dataset

It has been recognized that the fidelity and reproducibility of machine learning approaches are intrinsically linked to the integrity and quality of the underlying datasets. To enhance model performance, we introduced and focused on the physicochemical and PK properties of compounds when preparing the dataset in our previous work [45]. We demonstrated that incorporating a concentration-related category in the dataset reduces structural diversity, enhances model performance, and ultimately leads to more reliable predictions of drug–target interaction. Here we further considered and evaluated 3 categories: concentration, absorption, and elimination of PK properties. Regarding the concentration-related category, we considered AUC, Fb, and Vdss. Regarding the absorption-related category, we considered Papp and Tmax. Regarding the elimination-related category, we considered CLint and t_1/2_. Note that these PK properties were estimated using ADMET predictor^®^ ver. 8.0 [51] from Simulation Plus “https://www.simulations-plus.com (accessed on 15 October 2025)”. The dataset used in this study is the NCI-ALMANAC [52], which evaluated over 5000 combinations of 104 investigational and approved drugs. Synergy was assessed across 60 cancer cell lines, resulting in more than 290,000 synergy scores. The number of drug combination pairs for each cell line is presented in Table 2 (Section 2.2). The names of the drug combinations and their corresponding interaction types for the Huh-7 and HCT15 cell lines are provided in Tables S3 and S4, respectively. Regarding characteristics, we applied the 166-bit Molecular Access System (MACCS) [53] chemical fingerprinting techniques to characterize these compounds in terms of physicochemical properties (see the next section for details).

#### 4.1.2. Representation of Chemical Compounds

In chemoinformatics, fingerprints are essential binary representations used to indicate the presence or absence of specific molecular substructures or features. They encode various molecular properties, including atomic details, atomic surroundings, bond characteristics, and bond positioning. Their versatility makes them fundamental for predicting molecular behavior. Prominent fingerprinting methods include PubChem [54], the 166-bit Molecular Access System (MACCS) [53], and Extended-Connectivity Fingerprints (ECFPs) [55,56]. As demonstrated in our previous study [45], MACCS was identified as the most effective fingerprinting method and was therefore utilized in this study for the training dataset using PaDEL-Descriptor (version 2.2) [57].

#### 4.1.3. Heatmap Ranking Algorithm

**Figure 5:**
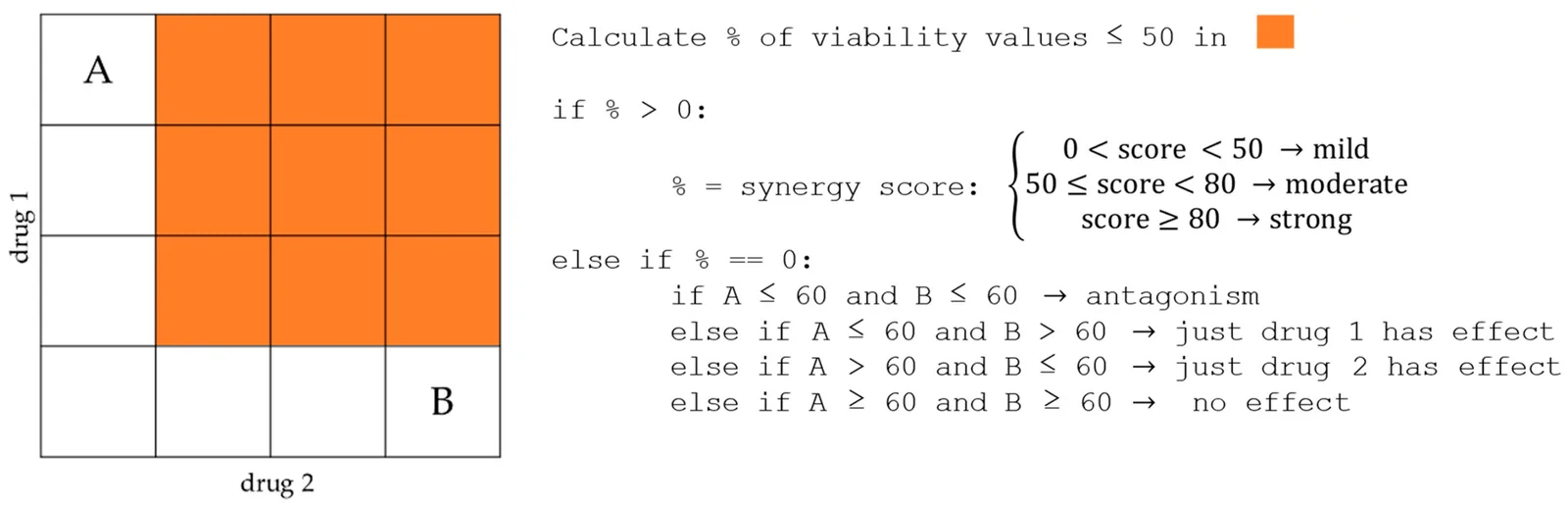
Pseudo-code for the decision rules implemented in the Python script to obtain an estimated synergy score based on the observation of the heatmaps for the combination effects tested in the NCI-ALMANAC study [44]. A represents the cell viability of Drug 1 alone at the highest concentration tested, and B represents the cell viability of Drug 2 alone at the highest concentration tested.

We utilized a straightforward method based on our previous work to classify the degree of synergism using pairwise dose–response heatmap data [44]. Figure 5 illustrates the decision rules applied in the algorithm. The heatmap in the figure represents cell viability percentages (ranging from 0% to 100%) after treatment with either the first drug (Drug 1), the second drug (Drug 2), or their combination at specific concentrations. First, the algorithm looks at the viability values in which both drugs are present at nonzero concentrations, shown in orange. From these nine orange-marked cells, it calculates the percentage of cells with viability at or below 50%. When this percentage is greater than zero, it is taken as a synergy score. According to empirical classifications of datasets, synergy is then divided into three levels:

- Mild when the score is below 50%.
- Moderate when the score falls between 50% and 80%.
- Strong when the score is above 80%.

If the synergy score is 0, indicating that the combination does not lead to a significant reduction in cell viability, additional parameters (A and B) are evaluated to assess whether the drug pair shows antagonism (i.e., at high concentrations the combination is less effective than either drug alone), whether the effect is mainly driven by one of the compounds, or whether no effect is present [44]. The single-cell data were obtained from the NCI-ALMANAC records using the corresponding plate number code associated with the combination data. It should be noted that, in several cases, discrepancies were identified between the single-drug viability values reported in the NCI-ALMANAC dataset and those displayed on the SYNERGxDB [41] platform. Consequently, the latter was considered less reliable than the synergy ranking, which showed strong consistency with evaluations based on visual inspection.

### 4.2. Machine Learning Models

#### 4.2.1. Classification Models

We extended the optimal ensemble-based ML model developed previously to predict the synergistic or antagonistic effects of drug pairs. In our previous work [45], we evaluated the impact of various base learner algorithms on prediction accuracy, considering two distinct ensemble stacking classification approaches. Our findings indicated that the optimal ensemble stacking classification model integrates Random Forest (RF), Gradient.

Boosting Machine (GBM), and Logistic Regression (LR) as base learners, with LR serving as the second/meta learner, achieving superior performance. Therefore, we adopted this method in the present study. The model building was conducted using Python 3.8 on the Compute Canada server. We used the Scikit-learn library package (version 0.18) [58] for conventional ML. We briefly introduce these individual classification algorithms:

Random Forest (RF) is an ensemble learning technique employed for both classification and regression tasks. It is particularly valued for its robustness to noise and superior accuracy compared to individual classifiers [59]. RF constructs an ensemble of decision trees, where each tree is trained on a bootstrapped subset of the training data, with consistent sample distributions across all trees. The final prediction is derived by majority voting from the individual trees. For an RF model consisting of NNN trees, the predicted class label *c* for a given instance *x* is determined by majority voting, as expressed in Equation (1).

(1) $$ı\left(x\right)={argmax}_{c}(\sum_{n=1}^{N}I_{h_{n}}\left(x\right)=c)$$

where $h_{n}$ is the nth tree of the RF, and *I* is the indicator function.

Logistic Regression (LR) is a linear model commonly used for classification tasks [60]. It models the relationship between the dependent (response) variable and one or more independent (explanatory) variables, quantifying the significance and strength of their influence on the response variable. The response variable represents the class label to be predicted, while the explanatory variables are the features used for prediction. LR outputs the probability that a given input belongs to a specific class, typically estimated using the logistic (sigmoid) function, as described in Equation (2).

(2) $$f\left(y\right)=\frac{ı}{1+e^{-k(y-y_{0)}}}$$

where *e* denotes the natural logarithm base, *l* denotes the curve’s maximum value, $y_{0}$ denotes the sigmoid midpoint’s y value, and *k* denotes the curve’s logistic growth rate or steepness.

Gradient Boosting Machine (GBM) is an ensemble learning technique that constructs a predictive model by sequentially adding weak learners, typically decision trees, to correct the errors of the combined ensemble [61]. Each new tree is trained to predict the residual errors of the preceding ensemble, effectively reducing bias and variance. The final prediction is the aggregate of all individual tree predictions.

The objective function Lin GBM is in Equation (3).

(3) $$L\;(\Theta )=\sum_{i=1}^{N}{\left(y_{i}-F(x_{i})\right)}^{2}+\sum_{t=1}^{T}\Omega (f_{t})$$

where *N* is the number of data points, $y_{i}$ is the true label for the i-th data point, $F(x_{i})$ is the predicted value for the i-th data point from the model, *T* is the number of trees, $f_{t}$ represents the t-th tree in the ensemble, and *Ω(ft)* is a regularization term to penalize the complexity of each tree (often a function of the number of nodes in the tree or the leaf values).

Ensemble stacking classification method is an advanced ensemble learning technique that combines multiple base models, often of diverse types, to improve predictive performance [62]. The core principle is to train several base learners on the training data independently. The outputs of these base models, which can include predictions or probabilities, are then used as inputs to a meta-model (second-level learner). The meta-model learns to optimally combine these predictions to produce a final output. This approach benefits from the complementary strengths of different models, effectively reducing both bias and variance, which enhances generalization ability. A stacking model typically involves base models such as decision trees, support vector machines, neural networks and gradient boosting machines, while the meta-model is commonly a linear model, such as logistic regression. The ensemble method is particularly effective when the base models exhibit different strengths, capturing varied aspects of the data’s underlying structure. Numerous studies have demonstrated that ensemble learning enhances prediction accuracy compared to individual models [63,64].

#### 4.2.2. Performance Metrics of Ensemble Stacking Classification Method

We used standard accuracy, precision, recall and F1-score, which are defined as Equations (4)–(7) below:

(4) $$Accuracy=\frac{TP+TN}{TP+TN+FP+FN}$$

(5) $$Precision=\frac{TP}{TP+FP}$$

(6) $$Recall=\frac{TP}{TP+FN}$$

(7) $$F1-score=\frac{2TP}{2TP+FP+FN}$$

where TP, FP, FN, and TN represent the number of true positives, false positives, false negatives, and true negatives, respectively, to evaluate model performance.

### 4.3. Proposed Ensemble Stacking Classification Model

In this study, the effects of compound pairs against cancer cells in the dataset were classified into two categories, synergistic and antagonistic, based on the heatmap ranking algorithm described in Section 4.1.3. Therefore, our goal is to develop an ensemble stacking interaction classification model to predict such effects. To ensure a balanced representation of each category, we set the least frequent effect class as the reference frequency and randomly excluded excess compounds from the other classes in each calculation for various cancer types and cell lines to avoid data bias.

The proposed model consists of four components as discussed here.

The first component is the preparation of the drug effects dataset. A Python script was developed to extract the SMILES structures of Compound A, Compound B, and their corresponding generic names from files obtained from the SYNER-GxDB software v2.2.4 tool [33], specifically from the NCI-ALMANAC [52] dataset. Note that this dataset includes data from various cancer cell lines, including HCT15 and HCT116 for colorectal cancer, Huh7 for liver cancer, BT-549 and HS 578T for breast cancer, and NCI-H460, NCI-H522, and A-549 for lung cancer.

The second component is the preparation of drugs’ PK data. As described in Section 4.1.1, we aim to investigate the impacts of drugs’ PK properties from three categories including concentration-, absorption- and elimination-related categories which are AUC, Fb, Vdss, Papp, Tmax, CLint and t_1/2_. These values were estimated for each compound pair based on their SMILES structures using ADMET Predictor^®^ version 8.0 (Simulation Plus, Research Triangle Park, NC, USA). The properties for drug 1 and drug 2 are designated as AUC_1_, Fb_1_, Vdss_1_, Papp_1_, Tmax_1_, CLint_1_ and t_1/21_, and AUC_2_, Fb_2_, Vdss_2_, Papp_2_, Tmax_2_, CLint_2_, and t_1/22_ respectively. Additionally, PaDEL-Descriptor was employed to generate MACCS fingerprints for the compound pairs, which were designated as MACCS1 and MACCS2, as detailed in Section 4.1.2. For each pair of compounds, we calculated the ratio of pharmacokinetic properties between drug 1 and drug 2, including AUC_1_/AUC_2_, Fb_1_/Fb_2_, Vdss_1_/Vdss_2_, Papp_1_/Papp_2_, Tmax_1_/Tmax_2_, CLint_1_/CLint_2_, and t_1/21_/t_1/22_. These ratios, together with molecular fingerprints (MACCS_1_ and MACCS_2_), were used as independent variables to predict the effects of compound pairs. We evaluated drug pairs using both the original (A + B) and reversed (B + A) representations to assess whether compound ordering affected model predictions. The compound-specific MACCS and pharmacokinetic features were exchanged accordingly. The predictions were unchanged after reversing the compound order, confirming that the model was not affected by the arbitrary ordering of the two compounds (Table S5).

The third component is the preparation of training and testing sets. To assess the robustness of the observed improvement associated with pharmacokinetic (PK) ratio features, we performed repeated nested cross-validation rather than relying exclusively on a single train–test split. The outer cross-validation loop was used to estimate the generalization performance of the model, while the inner loop was used exclusively for model selection and hyperparameter optimization. For each cell line and PK feature set, the nested cross-validation procedure was repeated 10 times using 5-fold outer cross-validation. Model performance was evaluated on each held-out outer test fold using accuracy in Table 2. To quantify uncertainty in model performance, 95% confidence intervals (CIs) were calculated.

Additionally, learning-curve analyses were performed as a complementary validation analysis to further assess the potential influence of sample size and the risk of overfitting. For each cell line and PK feature set, models were trained using progressively increasing fractions of the available training data, while maintaining the same model-development procedure used in the repeated nested cross-validation. Model performance was evaluated on held-out data at each training-set size. Training and validation performance were recorded for each training fraction and averaged across repeated resampling iterations. As shown in Figure S1, the learning-curve analysis of the ensemble stacking model for the HCT116 cell line dataset, using AUC_1_/AUC_2_ as independent variables, demonstrated a gradual decrease in training accuracy and an increase in validation accuracy as the training-set size increased. The validation accuracy approached the training accuracy, with no substantial divergence between the two, providing additional evidence of model stability with respect to training-set size.

In the following, to further assess model generalizability and minimize potential data leakage, we performed a drug-disjoint train–test split in addition to the original pair-random split. In this approach, individual drugs were separated into training and testing groups, ensuring that no drug appeared in both datasets through different combinations. The model was trained only on drug pairs involving compounds from the training group and evaluated on pairs involving previously unseen compounds. This provides a more stringent evaluation of whether the proposed pQSAR model can generalize to drugs not encountered during training.

The fourth component is the core of the proposed model, the stacking method. The Ensemble stacking classification algorithm in this work integrated RF, GBM, and LR as base learners, with LR serving as the second/meta learner. We employed empirical optimization for key hyperparameters such as n_estimators, random_state, and max_iter. This decision was supported by precedent from previous QSAR modeling studies, such as Kwon et al. [65], which have shown that empirically guided hyperparameter selection can yield robust and generalizable models. We set the number of estimators to 100 for RF and GBM based on experimental efficiency.

In terms of Ensemble stacking with a multilayer perceptron (MLP), a deep learning framework was implemented using an ensemble of MLP models, characterized by fully connected feedforward architectures, to perform binary classification. The MLP model was selected as a state-of-the-art baseline for comparison with the ensemble stacking approach due to its well-established capability to capture complex, nonlinear relationships in high-dimensional molecular descriptor spaces, a defining feature of cheminformatics and bioinformatics datasets [66].

## 5. Conclusions

Combination therapy is widely employed in cancer management to enhance therapeutic effectiveness, reduce adverse effects, and address the emergence of drug resistance. However, the large number of possible drug combinations makes experimental testing costly and time-consuming. Furthermore, because interactions between drugs may produce either synergistic or antagonistic effects, the development of reliable and efficient predictive models is essential.

In our previous work, we introduced and validated a simple empirical rule–based approach to estimate the degree of synergism from pairwise dose–response heatmap data [44]. In addition, we have demonstrated the applicability of the ensemble stacking method in predicting a single compound’s QSAR [45]. Building upon both foundations, therefore, we have developed a pairwise-compound quantitative structure–activity relationship (pQSAR) framework based on an ensemble stacking machine learning strategy to predict effects of cancer drug combinations against various cancer cell lines. By integrating molecular descriptors (MACCS fingerprints) with pharmacokinetic (PK) properties, we systematically evaluated the contribution of concentration- (AUC, Fb and Vdss), absorption- (Papp and Tmax), and elimination-related (CLint and t_1/2_) parameters to predictive performance using the NCI-ALMANAC dataset across eight cancer cell lines representing four cancer types. This dataset includes data from various cancer cell lines, including HCT15 and HCT116 for colorectal cancer, Huh7 for liver cancer, BT-549 and HS 578T for breast cancer, and NCI-H460, NCI-H522, and A-549 for lung cancer.

Our results demonstrate that the proposed ensemble stacking model, which combines RF, GBM, and LR with LR as the meta-learner, consistently outperforms individual models and alternative ensemble configurations. The model achieved accuracy values exceeding 0.88, with the highest performance observed when concentration-related PK ratio features were incorporated. Specifically, AUC represents the total drug exposure over time, Vdss reflects the extent of drug distribution into tissues, and bioavailability (Fb) reflects the fraction of administered drug that reaches systemic circulation.

A key finding of this work is that ratio-based pharmacokinetic descriptors (PK_1_/PK_2_ or PK_2_/PK_1_) significantly enhance predictive accuracy compared to using absolute PK values or molecular fingerprints alone. Notably, the highest predictive accuracy was achieved in Table 2 when AUC and Vdss were included, highlighting their central role in characterizing systemic exposure and drug distribution. These parameters directly reflect effective drug concentrations at target sites, which are critical determinants of synergistic and antagonistic interactions. In contrast, absorption and elimination-related properties (Papp, Tmax, CLint, and t_1_/_2_) showed comparatively smaller contributions to predictive performance. This suggests that relative exposure and distribution characteristics between paired drugs play a more decisive role in determining combination effects than isolated absorption or clearance metrics.

Overall, this study demonstrates that an ensemble stacking framework trained on data integrating concentration-related exposure metrics with molecular descriptors provides a computational framework for predicting experimentally observed drug combination-effect classes. By incorporating molecular and pharmacokinetic (PK)-related descriptors, the proposed approach provides predictive insights into drug combination effects without implying that it fully explains the underlying biological mechanisms of synergy or antagonism. The proposed pQSAR methodology provides a computational approach for predicting empirically defined drug-combination effects and may assist in prioritizing candidate synergistic drug pairs for subsequent experimental evaluation. However, independent external validation and prospective experimental studies are needed to establish its generalizability and utility for guiding combination-therapy development. In future work, we will extend the current framework by incorporating pharmacodynamic (PD) and cell-line-specific molecular descriptors to further improve the prediction of drug combination effects. This planned integration of PK–PD information will provide a more comprehensive and mechanistically informed framework for predicting cancer drug combination effects.

## Figures and Tables

**Table 1:** Performance comparison between the ensemble stacking classification methods and the individual models on the testing set of the HCT116 cell line dataset considering AUC_1_/AUC_2_ as independent variables.

ML Method	Accuracy	Precision	Recall	F1-Score
RF	0.75 ± 0.007	0.75 ± 0.006	0.75 ± 0.006	0.75 ± 0.006
GBM	0.76 ± 0.004	0.76 ± 0.008	0.75 ± 0.007	0.75 ± 0.009
LR	0.62 ± 0.01	0.62 ± 0.01	0.62 ± 0.01	0.62 ± 0.01
Ensemble stacking MLP (Ens-MLP)	0.84 ± 0.007	0.84 ± 0.008	0.83 ± 0.007	0.83 ± 0.008
Proposed Ensemble stacking-original pair-random split (P-Ens-org)	0.88 ± 0.005	0.87 ± 0.006	0.88 ± 0.005	0.88 ± 0.006
Proposed Ensemble stacking-drug-disjoint train–test split (P-Ens-dis)	0.80 ± 0.01	0.80 ± 0.01	0.80 ± 0.01	0.81 ± 0.01

**Table 2:** The accuracy scores of the testing set for the ensemble stacking classification method across four cancer types using eight representative cancer cell lines under three conditions: (i) using PK properties of individual drugs only, (ii) using no PK properties, and (iii) using PK ratio features (PK_1_/PK_2_ or PK_2_/PK_1_). The standard deviation (SD) and confidence interval (CI) are provided for each accuracy score considering AUC, Vdss and Fb. The last column presents the number of pairs for each cell line.

**Cell Lines**	**AUC_1_, AUC_2_**	**NO AUC**	**Ratio_1_ =** **(AUC1/AUC2)**	**Ratio_2_ =** **(AUC2/AUC1)**	**Number of Pairs**
Colorectal Cancer					
HCT15	0.83 ± 0.007 (0.82–0.84)	0.74 ± 0.009 (0.73–0.75)	0.92 ± 0.003 (0.91–0.93)	0.91 ± 0.003 (0.90–0.92)	31
HCT116	0.85 ± 0.004 (0.84–0.86)	0.63 ± 0.01 (0.61–0.64)	0.88 ± 0.008 (0.87–0.88)	0.88 ± 0.008 (0.87–0.88)	32
Liver Cancer					
Huh7	0.82 ± 0.007 (0.81–0.82)	0.72 ± 0.01 (0.71–0.73)	0.91 ± 0.003 (0.90–0.91)	0.90 ± 0.004 (0.89–0.90)	32
Breast Cancer					
BT-549	0.87 ± 0.007 (0.86–0.88)	0.73 ± 0.01 (0.72–0.74)	0.91 ± 0.003 (0.91–0.92)	0.90 ± 0.003 (0.89–0.90)	37
HS 578T	0.86 ± 0.009	0.76 ± 0.01	0.92 ± 0.004	0.90 ± 0.003	40
Lung Cancer	(0.85–0.87)	(0.75–0.77)	(0.91–0.92)	(0.89–0.90)	
NCI-H460	0.84 ± 0.003 (0.82–0.84)	0.76 ± 0.02 (0.75–0.77)	0.91 ± 0.005 (0.90–0.92)	0.90 ± 0.002 (0.89–0.92)	45
NCI-H522	0.85 ± 0.01 (0.83–0.85)	0.71 ± 0.02 (0.70–0.72)	0.90 ± 0.005 (0.89–0.90)	0.89 ± 0.002 (0.88–0.91)	44
A-549	0.84 ± 0.01 (0.83–0.85)	0.76 ± 0.02 (0.74–0.76)	0.91 ± 0.003 (0.90–0.91)	0.91 ± 0.003 (0.90–0.91)	42
**Cell Lines**	**Vdss_1_, Vdss_2_**	**NO Vdss**	**Ratio_1_** = **(Vdss_1_/Vdss_2_)**	**Ratio_2_** = **(Vdss_2_/Vdss_1_)**	**Number of Pairs**
Colorectal Cancer					
HCT15	0.80 ± 0.01 (0.79–0.81)	0.75 ± 0.02 (0.74–0.76)	0.93 ± 0.002 (0.92–0.93)	0.92 ± 0.002 (0.92–0.93)	31
HCT116	0.84 ± 0.01 (0.83–0.84)	0.76 ± 0.02 (0.75–0.77)	0.91 ± 0.002 (0.90–0.91)	0.90 ± 0.002 (0.90–0.91)	32
Liver Cancer					
Huh7	0.82 ± 0.01 (0.81–0.82)	0.76 ± 0.02 (0.75–0.77)	0.92 ± 0.002 (0.91–0.92)	0.91 ± 0.001 (0.90–0.91)	32
Breast Cancer					
BT-549	0.83 ± 0.01 (0.81–0.83)	0.74 ± 0.03 (0.73–0.76)	0.89 ± 0.006 (0.88–0.90)	0.89 ± 0.006 (0.88–0.90)	37
HS 578T	0.84 ± 0.009 (0.83–0.85)	0.78 ± 0.01 (0.77–0.79)	0.92 ± 0.006 (0.91–0.92)	0.91 ± 0.002 (0.90–0.92)	40
Lung Cancer					
NCI-H460	0.85 ± 0.009 (0.83–0.85)	0.78 ± 0.03 (0.77–0.80)	0.93 ± 0.004 (0.92–0.94)	0.92 ± 0.005 (0.91–0.93)	45
NCI-H522	0.80 ± 0.01 (0.79–0.81)	0.76 ± 0.03 (0.75–0.77)	0.89 ± 0.008 (0.88–0.91)	0.89 ± 0.009 (0.88–0.90)	44
A-549	0.85 ± 0.01 (0.84–0.86)	0.79 ± 0.03 (0.78–0.80)	0.92 ± 0.002 (0.91–0.92)	0.91 ± 0.003 (0.90–0.91)	42
**Cell Lines**	**Fb_1_, Fb_2_**	**NO F**	**Ratio_1_ =** **(Fb_1_/Fb_2_)**	**Ratio_2_ =** **(Fb_2_/Fb_1_)**	**Number of Pairs**
Colorectal Cancer					
HCT15	0.80 ± 0.01 (0.79–0.81)	0.74 ± 0.03 (0.73–0.75)	0.92 ± 0.002 (0.91–0.92)	0.92 ± 0.002 (0.91–0.92)	31
HCT116	0.82 ± 0.01 (0.80–0.82)	0.75 ± 0.03 (0.73–0.75)	0.90 ± 0.002 (0.89–0.90)	0.90 ± 0.002 (0.89–0.90)	32
Liver Cancer					
Huh7	0.80 ± 0.02 (0.78–0.81)	0.75 ± 0.02 (0.74–0.77)	0.90 ± 0.002 (0.89–0.90)	0.89 ± 0.003 (0.88–0.90)	32
Breast Cancer					
BT-549	0.82 ± 0.01 (0.80–0.82)	0.74 ± 0.03 (0.72–0.75)	0.89 ± 0.002 (0.88–0.90)	0.89 ± 0.002 (0.88–0.90)	37
HS 578T	0.81 ± 0.01 (0.80–0.82)	0.78 ± 0.02 (0.76–0.79)	0.90 ± 0.003 (0.89–0.90)	0.89 ± 0.002 (0.88–0.90)	40
Lung Cancer					
NCI-H460	0.83 ± 0.01 (0.82–0.84)	0.78 ± 0.02 (0.76–0.79)	0.90 ± 0.005 (0.89–0.90)	0.89 ± 0.002 (0.88–0.90)	45
NCI-H522	0.80 ± 0.02 (0.80–0.82)	0.77 ± 0.03 (0.76–0.78)	0.88 ± 0.005 (0.89–0.90)	0.88 ± 0.007 (0.87–0.90)	44
A-549	0.85 ± 0.02 (0.84–0.86)	0.77 ± 0.02 (0.76–0.78)	0.90 ± 0.003 (0.89–0.91)	0.90 ± 0.002 (0.89–0.90)	42
**Cell Lines**	**t_1/21_, t_1/22_**	**NO t_1/2_**	**Ratio_1_ =** **(t_1/21_/t_1/22_)**	**Ratio_2_ =** **(t_1/22_/t_1/21_)**	**Number of Pairs**
Colorectal Cancer					
HCT15	0.78	0.75	0.90	0.89	31
HCT116	0.79	0.76	0.88	0.87	32
Liver Cancer					
Huh7	0.78	0.76	0.89	0.89	32
Breast Cancer					
BT-549	0.80	0.74	0.84	0.84	37
HS 578T	0.81	0.78	0.86	0.85	40
Lung Cancer					
NCI-H460	0.80	0.78	0.89	0.88	45
NCI-H522	0.78	0.76	0.84	0.83	44
A-549	0.83	0.79	0.87	0.86	42
**Cell Lines**	**CLint_1_, CLint_2_**	**NO CLint**	**Ratio_1_ =** **(CLint_1_/CLint_2_)**	**Ratio_2_ =** **(CLint_2_/CLint_1_)**	**Number of Pairs**
Colorectal Cancer					
HCT15	0.77	0.73	0.89	0.88	31
HCT116	0.79	0.72	0.87	0.86	32
Liver Cancer					
Huh7	0.77	0.71	0.89	0.88	32
Breast Cancer					
BT-549	0.80	0.72	0.84	0.83	37
HS 578T	0.79	0.76	0.87	0.86	40
Lung Cancer					
NCI-H460	0.79	0.75	0.89	0.88	45
NCI-H522	0.77	0.75	0.84	0.84	44
A-549	0.81	0.74	0.87	0.86	42
**Cell Lines**	**Papp_1_, Papp_2_**	**NO Papp**	**Ratio_1_ =** **(Papp_1_/Papp_2_)**	**Ratio_2_ =** **(Papp_2_/Papp_1_)**	**Number of Pairs**
Colorectal Cancer					
HCT15	0.72	0.68	0.78	0.77	31
HCT116	0.69	0.67	0.76	0.75	32
Liver Cancer					
Huh7	0.71	0.67	0.77	0.76	32
Breast Cancer					
BT-549	0.69	0.64	0.75	0.74	37
HS 578T	0.72	0.67	0.77	0.77	40
Lung Cancer					
NCI-H460	0.73	0.68	0.78	0.77	45
NCI-H522	0.68	0.64	0.75	0.75	44
A-549	0.68	0.64	0.75	0.75	42
**Cell Lines**	**tmax_1_, tmax_2_**	**NO tmax**	**Ratio_1_ =** **(tmax_1_/tmax_2_)**	**Ratio_2_ =** **(tmax_2_/tmax_1_)**	**Number of Pairs**
Colorectal Cancer					
HCT15	0.74	0.69	0.81	0.80	31
HCT116	0.72	0.67	0.77	0.77	32
Liver Cancer					
Huh7	0.74	0.69	0.83	0.82	32
Breast Cancer					
BT-549	0.72	0.67	0.77	0.76	37
HS 578T	0.70	0.66	0.78	0.77	40
Lung Cancer					
NCI-H460	0.75	0.70	0.83	0.82	45
NCI-H522	0.74	0.67	0.81	0.80	44
A-549	0.75	0.68	0.78	0.77	42

## Data Availability

The datasets and codes supporting the conclusions of this article are available in: https://github.com/SV-JT/Synergism-of-Mixtures-of-Compounds- (access on 10 March 2026). All code will be fully accessible in a public repository after the completion of the required legal and administrative procedures. The datasets for the classification stacking method include MACCS fingerprints, Vdss1/Vdss2, and drug synergy data for the cell lines, provided in the CSV files Ratio-Cell line. The second dataset includes MACCS fingerprints, Vdss2/Vdss1, and drug synergy data for the same cell lines, provided in the CSV files Ratio-2-Cell line.

## Supplementary Materials

The following supporting information can be downloaded at: https://www.mdpi.com/article/10.3390/ijms27188345/s1.
