# Supplementary material for: A Machine Learning Approach for Predicting Synergism of Mixtures of Compounds in Treating Cancers
Source: Int J Mol Sci. 2026 Sep 19;27(18):8345. doi: 10.3390/ijms27188345 (PMC13607624; doi:10.3390/ijms27188345)
Supplement: Supplementary file 1 [file ijms-27-08345-s001.zip › ijms-4492349-supplementary.pdf]

Table S1. Summary of Parameters in applied ML models.

**Model**	**Hyperparameter**
Random Forest (RF)	n_estimators=100, random_state=42
Gradient Boosting Machine (GBM)	n_estimators=100, learning_rate=1.0, max_depth=1, random_state=42
Logistic Regression (LR)	max_iter=10000, random_state=42

n_estimators = The number of decision trees.

random_state= A parameter in train_test_split that controls the random number generator used to shuffle the data before splitting it.

learning_rate= How much a machine learning model adjusts its parameters at each step of its optimization algorithm.

max_depth= A parameter controls the maximum depth of the tree.

max_iter = A parameter that controls the maximum number of iterations the optimization algorithm will run to converge.

Table S2. Summary of Parameters in MLP Model

**Hyperparameter**	**Value(s) Used**
Hidden Layers	3 layers
Units per Layer	25, 50, and 25
Activation Function	relu (for hidden layers), sigmoid (for output)
Output Layer Units	1
Dropout Rate	0.1 in model3 and model4
Loss Function	binary_crossentropy
Optimizer	adam
Epochs	100

Table S3: Names of Drug Combinations and Their Interactions for Huh-7 Cell Line.

$Drug_1$	$Drug_2$	Drugs interaction
Apilimod	Clomifene citrate	Synergism
bepridil	TOREMIFENE CITRATE	Synergism
CHEMBL3348928	bepridil	Synergism
PIPERACETAZINE	Aripiprazole	Synergism
homoharringtonine	Sunitinib	Synergism
PIPERACETAZINE	Aripiprazole	Synergism
Sertraline hydrochloride	tetrandrine	Synergism
PIPERACETAZINE	Aripiprazole	Synergism
Sunitinib	PIPERACETAZINE	Synergism
Sertraline hydrochloride	PIPERACETAZINE	Synergism
Sertraline hydrochloride	tetrandrine	Synergism
Idrocilamide	Clomifene citrate	Synergism
colchicine	3-Deazaneplanocin	Synergism
colchicine	Sunitinib	Synergism
mycophenolate mofetil	Sunitinib	Antagonism
clomiphene	3-Deazaneplanocin	Antagonism
mycophenolate mofet	3-Deazaneplanocin	Antagonism
colchicine	mycophenolate mofetil	Antagonism
colchicine	TOREMIFENE CITRAT	Antagonism
homoharringtonine	PIPERACETAZINE	Synergism
bepridil	Sertraline hydrochloride	Synergism
clomiphene	Apilimod	Synergism
bepridil	TOREMIFENE CITRATE	Antagonism
Apilimod	Clomifene citrate	Antagonism
CHEMBL3348928	Sertraline hydrochloride	Synergism
CHEMBL3348928	Clomifene citrate	Synergism
Idrocilamide	bepridil	Synergism
colchicine	3-Deazaneplanocin	Antagonism
colchicine	homoharringtonine	Antagonism
mycophenolate mofetil	mycophenolate mofetil	Antagonism
colchicine	Sunitinib	Antagonism
Idrocilamide	Clomifene citrate	Antagonism

Table S4: Names of Drug Combinations and Their Interactions for HCT15 Cell Line.

$Drug_1$	$Drug_2$	Drugs interaction
sorafenib	cabazitaxel	Synergism
fluorouracil	vandetanib	Synergism
cabazitaxel	vismodegib	Synergism
azacitidine	sirolimus	Synergism
docetaxel	vismodegib	Synergism
mitomycin	oxaliplatin	Antagonism
topotecan	mitomycin	Antagonism
floxuridine	gemcitabine	Antagonism
cladribine	gemcitabine	Antagonism
pralatrexate	sirolimus	Synergism
azacitidine	cabazitaxel	Synergism
fluorouracil	cabazitaxel	Synergism

mitoxantrone	cabazitaxel	Synergism
sirolimus	cabazitaxel	Synergism
decitabine	pemetrexed	Synergism
sorafenib	cabazitaxel	Synergism
fluorouracil	Vandetanib	Synergism
dasatinib	sunitinib	Synergism
fluorouracil	sunitinib	Synergism
cabazitaxel	vismodegib	Synergism
erlotinib	topotecan	Synergism
cladribine	gemcitabine	Antagonism
floxuridine	gemcitabine	Antagonism
mitomycin	oxaliplatin	Antagonism
topotecan	mitomycin	Antagonism
dasatinib	pazopanib	Antagonism
ridaforolimus	dactolisib	Synergism
docetaxel	vismodegib	Synergism
vismodegib	pazopanib	Synergism
azacitidine	sirolimus	Synergism
vemurafenib	pazopanib	Synergism
vismodegib	pazopanib	Synergism

Table S5: Prediction consistency after reversing compound order on the testing set of the HCT116 cell line dataset considering AUC_1/AUC_2 as independent variables using ensemble stacking method

Drug pair	Input order	MACCS features	PK features	Accuracy
Drug A + Drug B	A → B	$MACCS_A$ / $MACCS_B$	PK_A / PK_B	0.88
Drug A + Drug B	B → A	$MACCS_B$ / $MACCS_A$	PK_B / PK_A	0.88

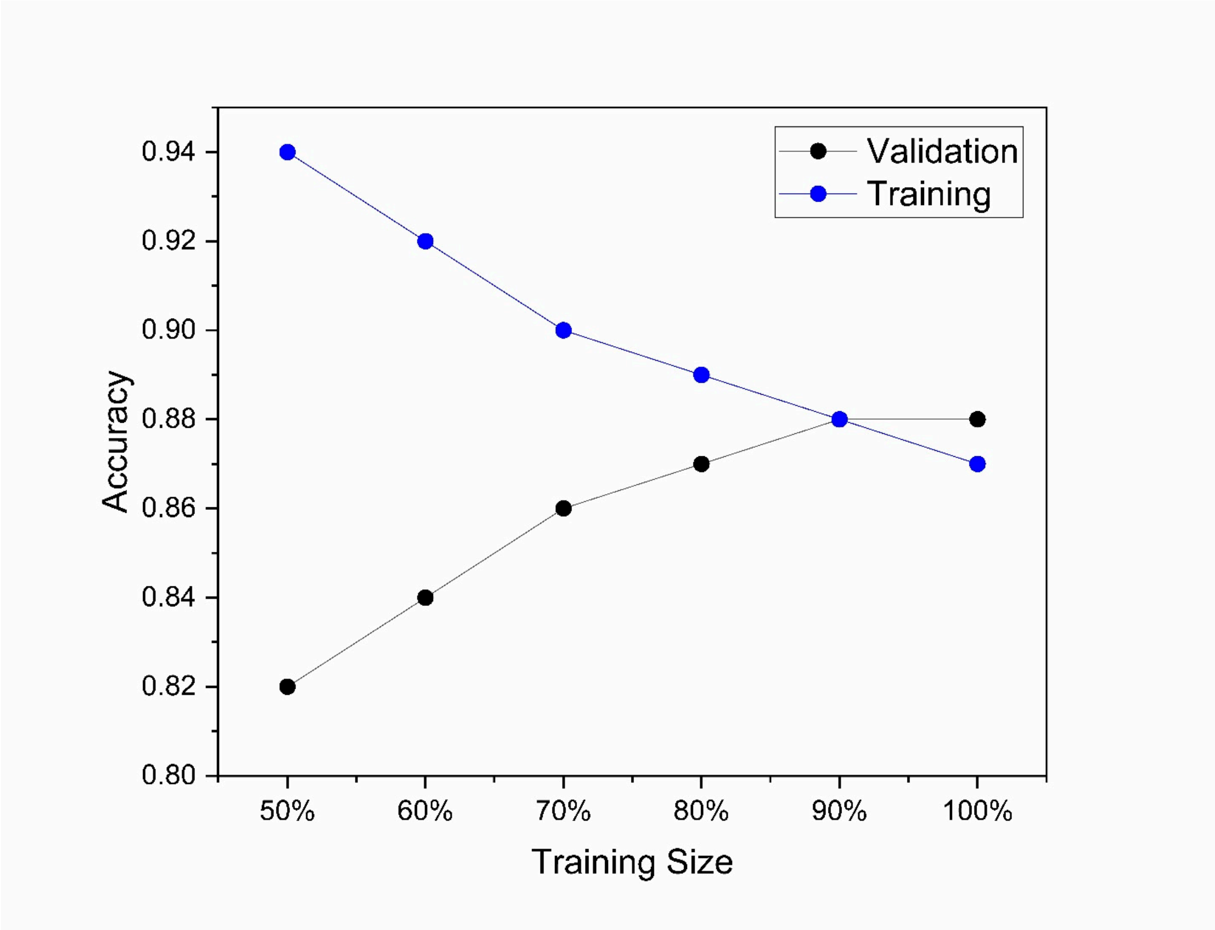

Figure S1. Learning curve analysis of the ensemble stacking model for the HCT116 cell line dataset using AUC_1/AUC_2 as independent variables.
